# Retrospective Study on the Efficacy and Safety of Pyrotinib-Based Therapy for HER2-Positive Nonbreast Advanced Solid Tumors

**DOI:** 10.1155/2022/4233782

**Published:** 2022-03-25

**Authors:** Jianzheng Wang, Baiwen Zhang, Xiaojiao Cheng, Qingli Li, Huifang Lv, Caiyun Nie, Beibei Chen, Weifeng Xu, Jing Zhao, Yunduan He, Shuiping Tu, Xiaobing Chen

**Affiliations:** ^1^Department of Medical Oncology, Affiliated Cancer Hospital of Zhengzhou University, Henan Cancer Hospital, Zhengzhou Henan Province 450008, China; ^2^Department of Oncology, Renji Hospital, School of Medicine, Shanghai Jiaotong University, Shanghai 200127, China

## Abstract

**Background:**

Human epidermal growth factor receptor 2 (HER2) is a member of the large ErbB family and an important oncogene in many solid tumors. Pyrotinib has been approved for the treatment of HER2-positive, recurrent, or metastatic breast cancer. However, there are very few clinical studies on pyrotinib in other HER2-positive solid tumors. Therefore, more evidence of clinical research is impendently needed to shepherd pyrotinib-based therapy in HER2-positive nonbreast advanced solid tumors. *Patients and Methods*. We performed a retrospective analysis of HER2-positive nonbreast advanced solid tumors patients with HER2 amplification or mutations who were administered with pyrotinib-based therapy in Henan Cancer Hospital between July 1, 2019, and December 2, 2021. In our research, 25 eligible patients were included with 16 patients with lung cancer, 6 patients with gastric cancer, 2 patients with colorectal cancer, and 1 patient with cholangiocarcinoma. Progression-free survival (PFS) is our main research end point.

**Results:**

The median PFS was 188 days (95% CI: 83–not reached (NR)), and overall survival (OS) was 250 days (95% CI: 188–NR), respectively. 16 patients with lung cancer had a median PFS of 204 days (95% CI: 55–NR) and 6 patients with gastric cancer had PFS of 142 days (95% CI: 83–NR), respectively. The median OS was 366 days (95% CI: 248–NR) in patients with lung cancer and 179 days (95% CI: 90–NR) in patients with gastric cancer. The median PFS and OS of patients receiving >3 line treatment were lower than those receiving ≤3 line treatment (PFS: 188 days vs 204 days, *p* = 0.92; OS: 188 days vs 366 days, *p* = 0.43). All 25 patients can be evaluated. The objective response rate (ORR) was 24%, and the disease control rate (DCR) was 68%. Lung cancer ORR was 25%, and gastric cancer ORR was 16.7%. In addition, the DCR of lung cancer was 62.5% and that of gastric cancer was 66.7%. In addition, the ORR and DCR of patients receiving treatment ≤3 lines were higher than those receiving treatment >3 lines (ORR: 35.7% vs 9.1%, *p* = 0.18; DCR: 71.4% vs 63.6%, *p* > 0.99). The most common treatment-related adverse events (TRAEs) were diarrhea (84%), but only 3 patients (12%) reported grade 3 diarrhea with good control.

**Conclusion:**

These results show that in HER2-positive nonbreast advanced solid tumors, the treatment based on pyrotinib regimen has good antitumor activity and acceptable safety. This retrospective study aims to promote larger clinical studies to further clarify the efficacy and safety of pyrotinib in the treatment of nonbreast solid tumors.

## 1. Introduction

Human epidermal growth factor receptor 2 (HER2) is a member of the ErbB receptor tyrosine kinase family. HER2 amplification, HER2 mutation, and overexpression of HER2 protein have been proved to be the main carcinogenic activation mechanisms [1]. HER2 positive is a key carcinogenic factor in about 15-20% of breast cancer. In the past twenty years, HER2-targeted therapy significantly improved the prognosis of early-stage and advanced HER2-positive breast cancer patients [2–4]. HER2 positive is also found in other solid tumors, such as lung squamous cell carcinoma, colon adenocarcinoma, and intrahepatic cholangiocarcinoma, and is considered an important predictor and prognostic marker of tumors [5, 6]. Although HER2-targeting drugs (trastuzumab, pertuzumab, ado-trastuzumab emtansine [TDM-1], and neratinib) have improved objective response rate (ORR) or survival in patients with HER2-positive stomach, lung, colorectal, cervical, endometrial, salivary, ovarian, and biliary cancers [7–10], primary and acquired resistance to HER2-targeted drugs largely limits its clinical application. Moreover, outside of breast and gastric cancer, the use of HER2 testing and therapy for amplification/overexpression/mutation remains controversial. Therefore, exploring effective HER2-targeted therapy is an unmet demand.

Pyrotinib is an oral irreversible pan-ErbB tyrosine kinase inhibitor (TKI) which can potently inhibit EGFR/HER1, HER2, and HER4. A number of clinical research have verified the efficacy of pyrotinib in the treatment of breast cancer. The regimen of pyrotinib combined with capecitabine has been approved for the treatment of HER2-positive recurrent or metastatic breast cancer in China [11, 12]. However, there are very few studies on pyrotinib in other HER2-positive solid tumors. This research explored the efficacy and safety of pyrotinib in the treatment of HER2-positive nonbreast advanced solid tumors.

## 2. Patients and Methods

### 2.1. Study Design, Therapeutic Regimen, and Efficacy Evaluation

Our research is observational, single center, and retrospective. 25 patients were recruited from Henan Cancer Hospital from July 1, 2019, to December 2, 2021. All patients were treated with pyrotinib-based treatment cycle for 21 days, and the treatment was stopped when unacceptable toxicity or disease progression occurred. Tumor imaging assessment was performed every two or three cycles according to the Response Evaluation Criteria in Solid Tumors (RECIST) version 1.1. All patients signed information consent forms before treatment.

### 2.2. Detection Standard of HER2 Alterations

Because of the different tumor types, the definitions of HER2-positive standards are different; we refer to the standards of previous clinical studies, gastric cancer, colorectal cancer, cholangiocarcinoma, and lung cancer HER2-positive diagnostic criteria; and the diagnostic methods were described. Gastric cancer: HER2-positive:immunohistochemistry (IHC)3+, or IHC2+ confirmed by Fluorescence in situ hybridization (FISH). If the tumor tissues are defined as HER2 in 10% or more tumor cells with the score of 3+ in the IHC method of uniform membrane staining, it is classified as HER2 positive [13]. Colorectal cancer and cholangiocarcinoma: tumors with 2+/3+ HER2 score in more than 50% of cells by IHC or with a HER2/CEP17 ratio higher than 2 in more than 50% of cells by FISH) [14, 15]. Lung cancer: according to the specimens including tumor tissue and plasma circulating tumor DNA (ctDNA), the changes of HER2 were identified by next-generation sequencing (NGS), and the amplification of HER2 was defined as the number of copies of HER2 (CN) ≥3.62 [16].

### 2.3. Evaluation of Efficacy and Detection of Adverse Events

The primary outcome is PFS, defined as the time from the onset of pyrotinib to disease progression or any cause of death. The secondary research endpoints include ORR, disease control rate (DCR), and overall survival (OS). OS was calculated from pyrotinib initiation until the date of death from any cause. Adverse events were collected from patients' medical records, and laboratory results were assessed in accordance with the National Cancer Institute Common Terminology Criteria for Adverse Events (NCI CTCAE) version 5.0.

### 2.4. Statistical Analysis

Statistical analyses were conducted using software GraphPad prism 8 and R 4.1.2 version. PFS and OS were analyzed using the Kaplan-Meier method. PFS between different subgroups were compared using the log-rank test (two-sided), and the corresponding 95% confidence intervals (CIs) were estimated using the Cox proportional regression model. Continuous variables were summarized using medians and ranges, and categorical variables were described using frequency and percentage. ORR and DCR between different subgroups were compared using the Fisher's exact test. *p* < 0.05 was considered to be statistically significant.

## 3. Results

### 3.1. Patient Characteristics

In this study, 25 eligible patients with advanced solid tumors with HER2 positive or HER2 mutation, excluding breast cancer patients, were enrolled and treated with pyrotinib-based treatment. The patient characteristics are summarized in Table 1. The median age was 56 (range 35–77 years), and 44% of the patients were male, and the median treatment line was 3. The 25 patients included 16 patients with lung cancer, 6 with gastric cancer, 2 with colorectal cancer, and 1 with cholangiocarcinoma. According to the methods of detection of HER2 alterations, IHC was used for assessing HER-2 status in 7 (28%) patients. FISH was used for 2 (8%) patients. Among the 16 lung cancer patients, the incidence of HER2 20 exon insertion mutation was the highest, with 12 patients, accounting for 48% of the total. There were 2 patients (8%) with exon20 p.Tyr772-Ala775dup, 1 patient (4%) with HER2 amplification, and 1 patient (4%) with HER2 p.D769Y. Eighteen (72%) patients received pyrotinib monotherapy as a third or further line of treatment. Treatment regimens were pyrotinib plus trastuzumab (2/25), pyrotinib combined with L-OHP chemotherapy drugs (1/25), pyrotinib combined with fruquintinib (1/25), pyrotinib combined with anlotinib and pembrolizumab (1/25), pyrotinib combined with nab-paclitaxel and trastuzumab (1/25), and pyrotinib combined with camrelizumab (1/25). Seventeen (68%) patients initiated pyrotinib treatment at 400 mg, 6 (24%) patients started with 320 mg, and 2 (8%) patients had a starting dose of 240 mg.

### 3.2. Efficacy

**Figure 1:**
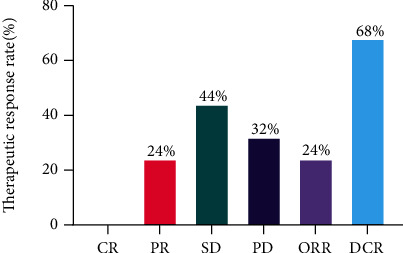
Tumor response in nonbreast advanced solid tumors.

**Figure 2:**
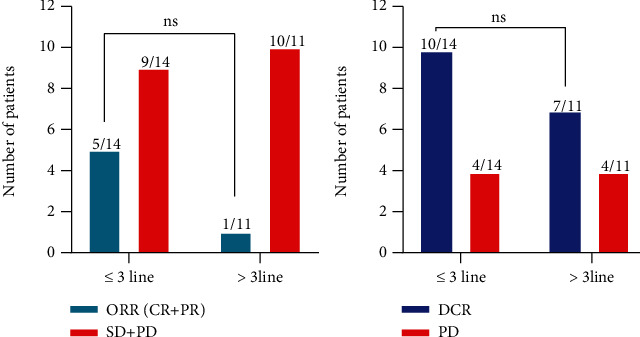
The ORR and DCR of patients (≤3line and >3lines). Patients receiving ≤3 lines of treatment had a numerically higher ORR and DCR than those receiving >3 lines of treatment (ORR: 35.7% vs 9.1%, *p* = 0.18; DCR: 71.4% vs 63.6%, *p* > 0.99); the difference was not statistically significant.

**Figure 3:**
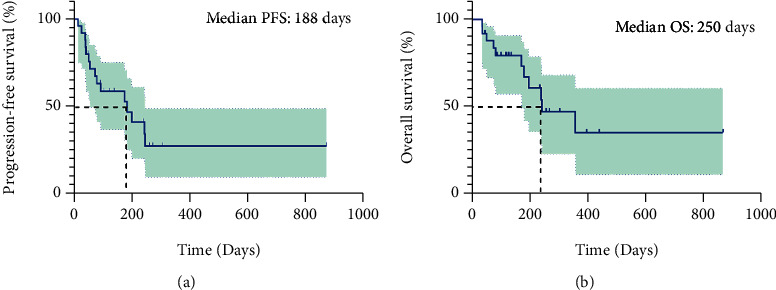
Kaplan-Meier curves of PFS (a) and OS (b) for all 25 patients with HER2-positive nonbreast advanced solid tumors.

**Figure 4:**
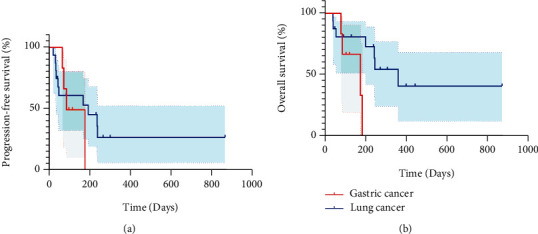
Kaplan-Meier curve of PFS (a) and OS (b) for 6 patients with gastric cancers and 16 patients with lung cancers.

**Figure 5:**
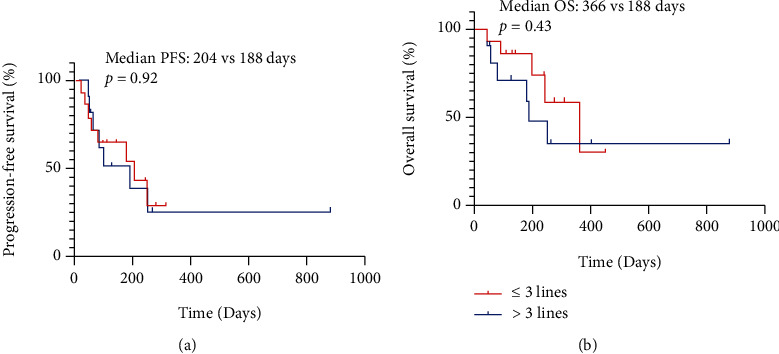
Kaplan-Meier curve of PFS (a) and OS (b) comparing ≤3 lines and >3 lines of pyrotinib-containing treatments.

The 25 patients available for efficacy evaluation included 16 with lung cancer, 6 with gastric cancer, 2 with colorectal cancer, and 1 with gallbladder cancer. 6 (24%) patients had PR, 11 (44%) patients achieved SD, and 8 (32%) patients had PD, resulting in an ORR of 24% and DCR of 68% (Figure 1). The best response of each patient is shown in Table 2. We performed subgroup analysis according to different tumor types, including lung cancer and gastric cancer. The ORR and DCR of different types of tumors treated with pyrotinib-based regimen are shown in Table 3.The ORR for lung cancer was 25% and for gastric cancer was 16.7%. In addition, the DCR for lung cancer was 62.5% and for gastric cancer was 66.7%. In addition, patients receiving ≤3 lines of treatment had a numerically higher ORR and DCR than those receiving >3 lines of treatment (ORR: 35.7% vs 9.1%, *p* = 0.18; DCR: 71.4% vs 63.6%, *p* > 0.99) (Table 4, Figure 2).The median PFS was 188 days (95% CI: 83–not reached (NR)), and overall survival (OS) was 250 days (95% CI: 188–NR), respectively (Figure 3). Sixteen patients with lung cancer had a median PFS of 204 days (95% CI: 55–NR), and six patients with gastric cancer had PFS of 142 days (95% CI: 83–NR), respectively (Figure 4(a)). The median OS was 366 days (95% CI: 248–NR) in patients with lung cancer and 179 days (95% CI: 90–NR) in patients with gastric cancer (Figure 4(b)). No statistical significance of a median PFS and OS was observed. The median PFS and OS of patients receiving >3 line treatment were lower than those receiving ≤3 line treatment (PFS: 188 days vs 204 days, *p* = 0.92; OS: 188 days vs 366 days, *p* = 0.43) (Figures 5(a) and 5(b)).

### 3.3. Safety

All of the 25 patients experienced some treatment-related adverse events (TRAEs), and most of these were grade 1 or 2. No TRAE of grade 4 or 5 was reported. The most common TRAEs were diarrhea (84%), but only 3 (12%) patients reported grade 3 diarrhea which was well controlled. Other TRAEs included asthenia (65%), nausea (48%), mucositis (40%), vomiting (32%), and hypertension (28%). TRAEs (≤20%) included rash (16%), hand-foot syndrome (24%), abdominal pain (12%), anemia (12%), and leukopenia (12%), and all were grade 1 or 2. There were no deaths related to pyrotinib treatment (Table 5).

## 4. Discussion

Tumor therapy has entered the era of precision treatment. Clinicians should perform relevant genetic testing before treatment, so as to choose the appropriate therapeutic plan for patients. Molecular typing-guided therapy has been applied in routine clinical practice. Genetic testing for advanced nonsmall cell lung cancer (NSCLC) includes EGFR, ALK, ROS1, BRAF, MET, HER-2, RET, NTRK1/2/3, PI3K, and PD-L1. HER2 and EGFR are members of the EGFR family; both of them are proliferation-driven genes of NSCLC. At present, there is no effective tyrosine kinase inhibitor (TKI) standard treatment recommendation for HER2 mutant NSCLC, and effective therapy for such population is one of the problems in the field of cancer. HER2 is a proliferation-driven gene, which has amplification, overexpression, and mutation in cancers. HER2 mutation accounted for 1%–2% in NSCLC. Most NSCLC patients with HER-2 gene mutation are women, nonsmokers, and adenocarcinoma. The main mutation form was exon 20 insertion mutation [17–21]. The National Medical Products Administration (NMPA) has approved the application of pyrotinib combined with capecitabine in HER2-positive advanced breast cancer, but pyrotinib has not yet been for advanced nonsmall cell lung cancer. For the treatment of HER2-mutated NSCLC, DS-8201 and T-DM1 are currently recommended in NCCN guidelines [22, 23]. Other TKI drugs are not recommended because there is no high-level evidence. Although mobocertinib (TAK-788), poziotinib, and other drugs have been reported as the latest data at international conferences, ORR is about 30%, but because these drugs are not listed in the domestic market, it is not available in China.

Pyrotinib is a small molecule TKI drug developed independently in China. As a novel oral irreversible TKI of pan-HER family, pyrotinib can prevent the formation of heterodimer of HER2 in tumor cells by covalently binding to ATP binding sites in the intracellular tyrosine kinase domain, inhibit its phosphorylation, and block the activation of downstream signaling pathways, thereby inhibiting tumor cell growth [24]. For advanced NSCLC with HER2 mutation, only two phase II studies in China found that pyrotinib showed good efficacy [25, 26]. For HER2-amplified advanced NSCLC, a small-scale prospective phase II clinical study published recently confirmed that pyrotinib has good efficacy and controllable safety. Due to the low frequency of HER2 amplification, this study included only 27 patients with HER2 amplification [27]. A retrospective real-world study (PEARL) demonstrates that pyrotinib-based therapy has good antitumor effects and an acceptable safety profile in NSCLC with heterogeneous HER2 alterations [28]. So far, there are no large-scale phase III clinical studies to further confirm the efficacy of pyrotinib in HER2-positive advanced NSCLC. Zhou et al. reported that the effective rate of pyrotinib monotherapy was 30% in HER2-mutant advanced lung adenocarcinoma. The ORRs were similar between patients with and without brain metastases (25.0% v 31.3%) [25]. Lu et al. reported that the effective rate of pyrotinib monotherapy was 22.2% in HER2-amplified advanced NSCLC. In our study, the ORR of pyrotinib in NSCLC was 25%, while the proportion of pyrotinib monotherapy was as high as 93.75% (15/16). Therefore, our research results are consistent with those of Zhou and Lu. Yang et al. reported that the ORR is 45.5% when pyrotinib is combined with apatinib for HER2-mutant or amplified metastatic NSCLC [26]. Yin et al. reported that the ORR is 44.4%; however, in this study, more than half of lung cancer patients adopt the strategy of pyrotinib combination [29]. These results suggest that pyrotinib should be used in combination in HER2-positive advanced NSCLC. PFS is 6.9 months in Zhou's study, 6.3 months in Lu's study, and 6.8 months in Yin's reports. In our study, PFS is 204 days (6.8 months) in lung cancer. Our results are highly consistent with those findings.

For HER2-positive advanced gastric cancer patients, trastuzumab is currently considered a first-line standard therapy, but for second-line and later treatment, there is currently no standard regimen. The Chinese Society of Clinical Oncology (CSCO) guideline (2021 version) recommends that clinical studies be encouraged (level III recommendations). Other HER2-targeted drugs such as pertuzumab (antiHER2 monoclonal antibody) and lapatinib (small molecule tyrosine kinase inhibitor) all ended in failure [30–32]. Although the results of the phase III clinical study on the antibody drug conjugate (ADC) TDM-1 in second-line treatment of gastric cancer were negative [33], ADC drugs with HER2 continued to receive attention. The results of phase II study showed that DS8201 and RC48 had good tumor response rate and survival benefit for patients with advanced HER2-positive gastric cancer who failed trastuzumab treatment [34, 35]. Therefore, effective HER2-targeted therapy for these patients is an unmet need, and efforts are being made to develop new antiHER2 drugs. Pyrotinib is a potent choice for patients who progress after receiving trastuzumab. The ORR and DCR of apatinib in the third line of gastric cancer were 1.7% and 31.82%, respectively [36]. In our study, the ORR and DCR of pyrotinib-based regimen were 16.7% and 66.7%, respectively, which is an encouraging result.

For HER2-positive colorectal cancer, there is a lack of relevant data on antiHER2-targeted therapy in China. Referring to the NCCN guidelines (2021 version), it is recommended that trastuzumab + pertuzumab or trastuzumab + lapatinib be used for the third-line treatment of advanced colorectal cancer with HER2 amplification [7, 14]. The aim of HERACLES-B trial is to assess the efficacy of the combination of pertuzumab and trastuzumab-emtansine (T-DM1) in HER2-amplified metastatic colorectal cancer; the trial did not reach its primary end point of ORR. However, based on the high disease control, similar PFS to other antiHER2 regimens, and low toxicity, pertuzumab in combination with T-DM1 can be considered for HER2 + mCRC as a potential therapeutic resource [37]. In our study, there are only two cases of advanced colon cancer, so we cannot determine whether pyrotinib benefited these patients. We will continue to explore the efficacy of pyrotinib in HER2-positive colon cancer in future studies.

Biliary carcinoma (BTCs) is a group of solid tumors with poor prognosis, and the treatment plan is limited. Chemotherapy based on gemcitabine combined with cisplatin has always been the preferred systematic treatment for biliary tumors [38]. In terms of therapeutic application, so far, there is no randomized controlled trial showing the efficacy of antiHER2 drugs in the field of biliary tract tumors. In fact, all evidence of their activity in these tumors is retrospective, or at an early stage of development, such as preclinical (in vitro and in vivo models) or from phase I trials [39].

In ASCO2020, Bob reported on the safety and efficacy of pyrotinib in patients with NSCLC and other solid tumors. The ORR was 19% (8/42, 95% CI 7-31%); confirmed responses include a complete response (CR) and 3 partial responses (PRs) in HER2-mutant NSCLC and 4 PRs in HER2-amplified cholangiocarcinoma, ovarian, endometrial, and salivary gland carcinomas [40]. Pyrotinib is the first TKI with persistent response in HER2-amplified biliary, ovarian, endometrial, and salivary gland cancers. In our study, the HER2-positive advanced cholangiocarcinoma patient achieved PR efficacy and was followed up to December 2, 2021. The PFS has reached 142 days and is still in treatment.

This study has certain limitations. First, this is a retrospective study that may have patient information bias and potential data missing; second, the sample size of this study is small; the results need to be further confirmed in larger samples. Additionally, not all patients with HER2-positive cancers were benefited from treatment with HER2-targeted therapies. Thus, more precise selection of patients to receive HER2-targeted therapies is required.

## 5. Conclusion

Extending the clinical benefits of HER2-targeted therapies beyond breast cancer to other HER2-positive solid tumors is an area of active investigation. In this study, we showed that pyrotinib-based therapy has promising antitumor activity and an acceptable safety profile in nonbreast HER2-positive advanced solid tumors.

## Figures and Tables

**Table 1:** Demographic and clinicopathologic characteristics of patients.

Characteristics	*N* = 25
Median age of patients, years (range)	56 (35-77)
≤60, *n* (%)	16 (64%)
>60, *n* (%)	9 (36%)
Gender, *n* (%)	
Male	11 (44%)
Female	14 (56%)
ECOG performance status, *n* (%)	
0-1	9 (36%)
≥2	16 (64%)
Tumor type	
Lung cancer	16 (64%)
Gastric cancer	6 (24%)
Colorectal cancer	2 (8%)
Cholangiocarcinoma	1 (4%)
Line of pyrotinib treatment, *n* (%)	
1	2 (8%)
2	9 (36%)
3	3 (12%)
4	5 (20%)
≥5	6 (24%)
Median (range)	3 (1-9)
Pyrotinib treatment regimen, *n* (%)	
Pyrotinib	18 (72%)
Pyrotinib + trastuzumab	2 (8%)
Pyrotinib + L-OHP	1 (4%)
Pyrotinib + fruquintinib	1 (4%)
Pyrotinib+ anlotinib + pembrolizumab	1 (4%)
Pyrotinib+ nab-paclitaxel + trastuzumab	1 (4%)
Pyrotinib + camrelizumab	1 (4%)
HER-2 status type, *n* (%)	
IHC3+	7 (28%)
FISH+	2 (8%)
Amplification	1 (4%)
exon20 insertion mutations	12 (48%)
exon20 p.Tyr772-Ala775dup	2 (8%)
p.D769Y	1 (4%)
Starting dosage of pyrotinib, *n* (%)	
400 mg	17(68%)
320 mg	6 (24%)
240 mg	2 (8%)

**Table 2:** Tumor response.

Best response	*N* = 25, *n* (%)
CR	0 (0%)
PR	6 (24%)
SD	11(44%)
PD	8 (32%)
ORR	6 (24%)
DCR	17(68%)

**Table 3:** Tumor response in lung cancer and gastric cancer.

Best response	Lung cancer	Gastric cancer
CR, *n*	0	0
PR, *n*	4	1
SD, *n*	6	3
PD, *n*	6	2
ORR, *n* (%)	25%	16.7%
DCR, *n* (%)	62.5%	66.7%

**Table 4:** Tumor response in patients receiving ≤3 lines and >3 lines treatment.

Best response	≤3 lines	>3 lines
CR, *n*	0	0
PR, *n*	5	1
SD, *n*	5	6
PD, *n*	4	4
ORR, *n* (%)	35.7%	9.1%
DCR, *n* (%)	71.4%	63.6%

**Table 5:** Treatment-related adverse events that occurred in at least 10% of patients.

AE	All grade, *n* (%)	Grade ≥ 3, *n* (%)
Diarrhea	21 (84)	3 (12)
Asthenia	17 (65)	2 (8)
Nausea	12 (48)	1 (4)
Mucositis	10 (40)	1 (4)
Vomiting	8 (32)	1 (4)
Hypertension	7 (28)	0
Rash	4 (16)	0
Hand-foot syndrome	4 (16)	0
Abdominal pain	3 (12)	0
Anemia	3 (12)	0
Leukopenia	3 (12)	0

## Data Availability

There are no underlying data that support the results of the study.
